# Radiation Therapy and Cardiac Death in Long-Term Survivors of Esophageal Cancer: An Analysis of the Surveillance, Epidemiology, and End Result Database

**DOI:** 10.1371/journal.pone.0158916

**Published:** 2016-07-18

**Authors:** Laila Gharzai, Vivek Verma, Kyle A. Denniston, Abhijeet R. Bhirud, Nathan R. Bennion, Chi Lin

**Affiliations:** 1 College of Medicine, University of Nebraska Medical Center, Omaha, NE, United States of America; 2 Department of Radiation Oncology, University of Nebraska Medical Center, Omaha, NE, United States of America; University Hospital Oldenburg, GERMANY

## Abstract

**Objective:**

Radiation therapy (RT) for esophageal cancer often results in unintended radiation doses delivered to the heart owing to anatomic proximity. Using the Surveillance, Epidemiology, and End Results (SEER) database, we examined late cardiac death in survivors of esophageal cancer that had or had not received RT.

**Methods:**

5,630 patients were identified that were diagnosed with esophageal squamous cell carcinoma (SCC) or adenocarcinoma (AC) from 1973–2012, who were followed for at least 5 years after therapy. Examined risk factors for cardiac death included age (≤55/56-65/66-75/>75), gender, race (white/non-white), stage (local/regional/distant), histology (SCC/AC), esophageal location (<18cm/18-24cm/25-32cm/33-40cm from incisors), diagnosis year (1973-1992/1993-2002/2003-2012), and receipt of surgery and/or RT. Time to cardiac death was evaluated using the Kaplan-Meier method. A Cox model was used to evaluate risk factors for cardiac death in propensity score matched data.

**Results:**

Patients who received RT were younger, diagnosed more recently, had more advanced disease, SCC histology, and no surgery. The RT group had higher risk of cardiac death than the no-RT group (log-rank p<0.0001). The median time to cardiac death in the RT group was 289 months (95% CI, 255–367) and was not reached in the no-RT group. The probability of cardiac death increased with age and decreased with diagnosis year, and this trend was more pronounced in the RT group. Multivariate analysis found RT to be associated with higher probability of cardiac death (OR 1.23, 95% CI 1.03–1.47, HR 1.961, 95% CI 1.466–2.624). Lower esophageal subsite (33–40 cm) was also associated with a higher risk of cardiac death. Other variables were not associated with cardiac death.

**Conclusions:**

Recognizing the limitations of a SEER analysis including lack of comorbidity accountability, these data should prompt more definitive study as to whether a possible associative effect of RT on cardiac death could potentially be a causative effect.

## Introduction

Esophageal cancer is the eighth most commonly diagnosed malignancy worldwide and the sixth leading cause of cancer death [1]. The overall incidence of esophageal cancer has increased slowly over time; specifically, the adenocarcinoma subtype has seen a four hundred percent rise in incidence since the 1970s [2]. Definitive surgery alone is the treatment of choice for patients with early-stage/localized and non-nodal disease; locally advanced (>T2 or N+) disease is treated with neoadjuvant concurrent chemoradiation, proceeding to surgery if operable [3].

Despite multi-modality treatment, overall five-year survival is poor, with only 18% of patients surviving five years from diagnosis [4]. Although mortality and morbidity are often directly related to disease complications, treatment can also incur significant morbidity. Radiation therapy (RT) can produce acute toxicities, which largely self-remit, but can also lead to more chronic toxicities. These late effects of RT are not well studied in diseases with poorer prognoses owing to poor survival. Anatomic proximity of the esophagus to the heart, lungs, and other regions of the esophagus, can cause chronic toxicities in these organs that can compromise quality of life (and potentially survival) in esophageal cancer survivors.

Specifically, RT-associated cardiac injury in esophageal cancer has not been well explored. The cellular basis of radiation damage has been well studied. Microvascular injury [5] and fibrosis [6] secondary to radiation exposure leads to accelerated atherosclerosis, which has also been demonstrated by a mouse model [7]. Late effects of RT can manifest as congestive heart failure, ischemia, coronary artery disease, valvular disease, or myocardial infarction, all potential causes of cardiac death.

This issue is being increasingly documented in survivors of neoplasms with good prognoses such as Hodgkin’s lymphoma and breast cancer. Regarding the former, the now-outdated use of comprehensive thoracic lymphatic irradiation (utilizing the so-called “mantle field”) has resulted in estimated relative risks of cardiac morbidity/mortality of 2–42 times above baseline [8,9,10,11] Additionally in left-sided breast cancer, recent data have demonstrated a 7.4% increased risk of cardiac events per Gray of mean heart RT dose [12], a value that could be higher when accounting for preexisting comorbidities [13].

In this study, we utilize the Surveillance, Epidemiology, and End Results (SEER) database to examine late cardiac death in esophageal cancer patients receiving RT. Although a causal relationship cannot be implied from these data, this warrants further assessments of possible cardiac ramifications from esophageal cancer RT.

## Materials and Methods

### Patient Selection

A total of 48,898 patients diagnosed with esophageal (site code: C150-C159) squamous cell carcinoma (histology code: 8070–8075) (SCC) or adenocarcinoma (histology code: 8140–8143, 8480–8481) (AC) between 1973 and 2012 with at least 5-year follow up were identified from the SEER database. After excluding records with missing data on RT, 47,764 patients remained. To identify patients at risk for late cardiac mortality, only patients who survived for five or more years were identified for inclusion, leaving a study population of 5,630 patients. For the purposes of our study, cardiac death was defined as death from diseases of the heart (recode: 50060). Salient risk factors were then examined, including age, gender, race, histology, esophageal subsite, stage, diagnosis year, and treatment modality (RT and surgery) (Table 1). Age was stratified into four groups: less than or equal to 55, 56–65, 66–75, and greater than 75. Disease subsite was grouped into four sections, based on distance from the incisors: 15–18 cm (site code: C150), 19–24 cm (site code: C151-C153), 25–32 cm (site code: C154), and 33–40 cm (site code: C155). The SEER stage of disease was classified as local, regional, or distant. Diagnosis year was divided into 3 periods: 2003–2012, 1993–2002, and 1973–1992. We chose to examine until 2012 owing to the publication of the landmark phase III CROSS trial in 2012 showing large magnitudes of survival benefits (median overall survival doubling from 24 months to 49 months) using preoperative carboplatin/paclitaxel-based chemoradiation (41.4 Gy) followed by surgery. Thereafter, this caused a major paradigm shift in the treatment of esophageal cancer [3].

**Table 1:** Clinical characteristics of the study population.

	RT N (%)	No RT N (%)	P Value (Chi-square)
	N = 3014	N = 2616	
Age (year)			**0.008**
Median	64	65	
≤ 55	754 (25)	568 (22)	
56–65	956 (32)	815 (31)	
66–75	901 (30)	837 (32)	
> 75	403 (13)	396 (15)	
Race			**< 0.0001**
White	2462 (82)	2289 (87.5)	
Non-White	552 (18)	327 (12.5)	
Gender			**< 0.0001**
Male	2140 (71)	2038 (78)	
Female	874 (29)	578 (22)	
Histology			**< 0.0001**
SCC	1695 (56)	805 (31)	
AC	1319 (44)	1811 (69)	
Site			**< 0.0001**
15–18	148 (5)	41 (1.5)	
19–24	393 (13)	171 (6.5)	
25–32	634 (21)	364 (14)	
33–40	1536 (51)	1653 (63)	
unknown	303 (10)	387 (15)	
Stage			**<0.0001**
Local	1210 (40)	1863 (71)	
Regional	1218 (40)	448 (17)	
Distant	255 (9)	74 (3)	
Unknown	331 (11)	231 (9)	
Year of diagnosis			0.054
1973–1992	569 (19)	561 (21)	
1993–2002	1151 (38)	959 (37)	
2003–2012	1294 (43)	1096 (42)	
Surgery			**<0.0001**
Yes	1425 (47.3)	2071 (79.2)	
No	1582 (52.5)	531 (20.3)	
Unknown	7 (0.2)	14 (0.5)	
Total cardiac death			0.2
Yes	332 (11)	262 (10)	
No	2682 (89)	2354 (90)	

Statistically significant values are in bold. RT, radiotherapy; SCC, squamous cell carcinoma; AC, adenocarcinoma.

### Statistical Methods

Descriptive statistics were used in reporting the demographics and disease characteristics by two groups (no-RT and RT), and chi-squared tests were used for comparison between groups. Patient populations were also matched by propensity score, which was designed to control for confounding variables and examine outcomes in this controlled, matched population.

Time to cardiac death was evaluated using the Kaplan-Meier method. Multivariate logistic regression was used to model the likelihood of cardiac death per group between 1973 and 2012 while controlling for patients’ age and the year of diagnosis. Odds ratios (OR) and 95% confidence intervals (CI) were computed using PROC LOGISTIC in SAS. Cox proportional hazards regression was used to assess hazard ratios (HR). All statistical calculations were performed using SAS version 9.4 (SAS Institute Inc., Cary, NC). Statistical significance was set at P < 0.05.

## Results

### Patient and Tumor Characteristics

The 5,630 analyzed patients were predominantly male (75%) and white (85%). Patients tended to be young, with only 13.5% older than 75 years. Approximately 42% of the patients had SCC, and 58% AC. Additionally, distal disease was most common, with 86% of tumors occurring greater than 25 cm from the incisors. Isolated local disease was diagnosed in 60% of patients, 34% had regional involvement, and 7% had distant metastases. Patients also tended to be diagnosed more recently, with 44% of patients diagnosed in 2003 or later. Finally, 68% of patients underwent surgery and 54% received RT. Patients who received RT tended to be younger, diagnosed more recently, have more advanced disease, SCC histology & more proximal disease, and had not undergone surgery (Table 1).

### Survival Analysis

**Fig 1:**
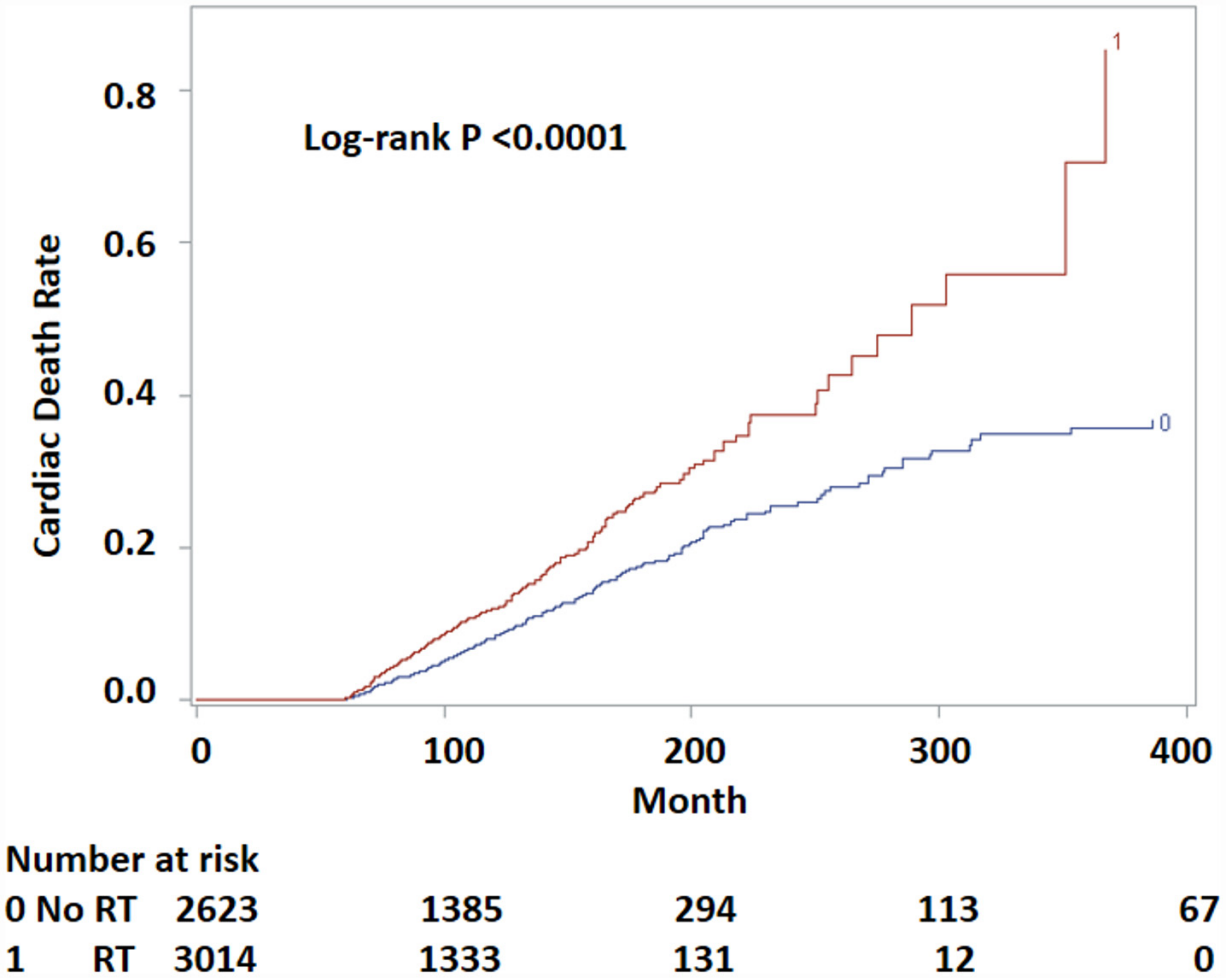
Probability of cardiac death by RT status for long-term survivors of esophageal cancer. Numbers at risk indicate patients liable to be censored or undergo failure at each time period.

**Fig 2:**
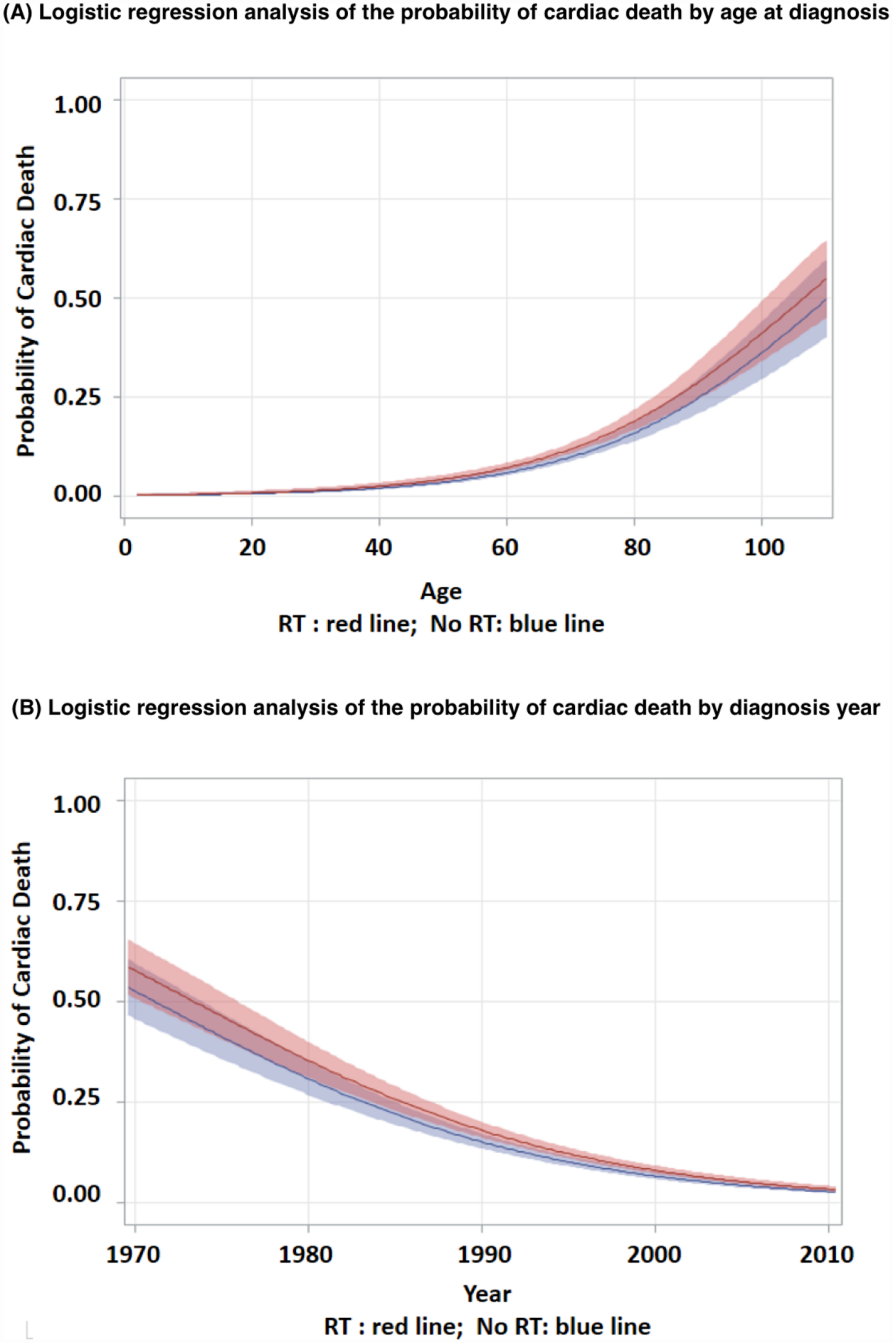
Logistic regression analysis of the probability of cardiac death by (A) age at diagnosis and (B) diagnosis year (shading represents 95% confidence interval).

**Fig 3:**
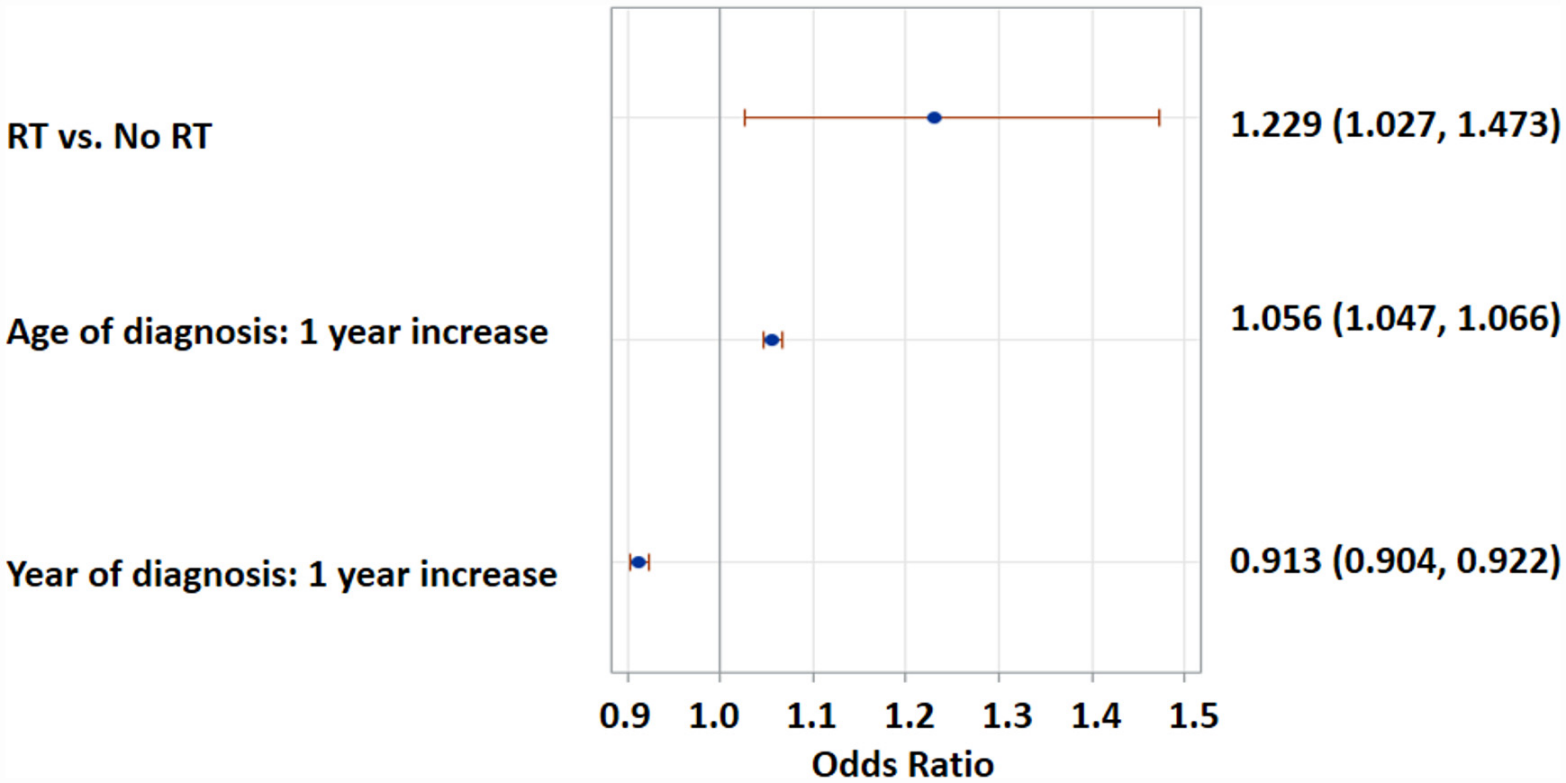
Multivariate logistic regression analysis of the probability of cardiac death by RT, adjusted by age at diagnosis and diagnosis year.

Patients who received RT had a higher risk of cardiac death than those who did not (log-rank p < 0.0001, Fig 1). The median time to cardiac death in patients receiving RT was 289 months (95% CI, 255–367), whereas this time point was not reached in the no-RT group. The probability of cardiac death increased with age and decreased with diagnosis year (Fig 2). This trend was more pronounced in the RT group. Multivariate logistic regression analysis found RT to be significantly associated with a 22% higher probability of cardiac death than no-RT (OR 1.23; 95% CI, 1.03–1.55), after adjusting for age and diagnosis year (Fig 3). Gender, race, histology, stage, esophageal subsite and surgery status were not associated with cardiac death. The probability of cardiac death could be predicted by the following equation generated from the above multivariate regression model: 179.7 + 0.2345*RT -0.0929*year + 0.0544*age.

### Propensity Score Matching Analysis

**Fig 4:**
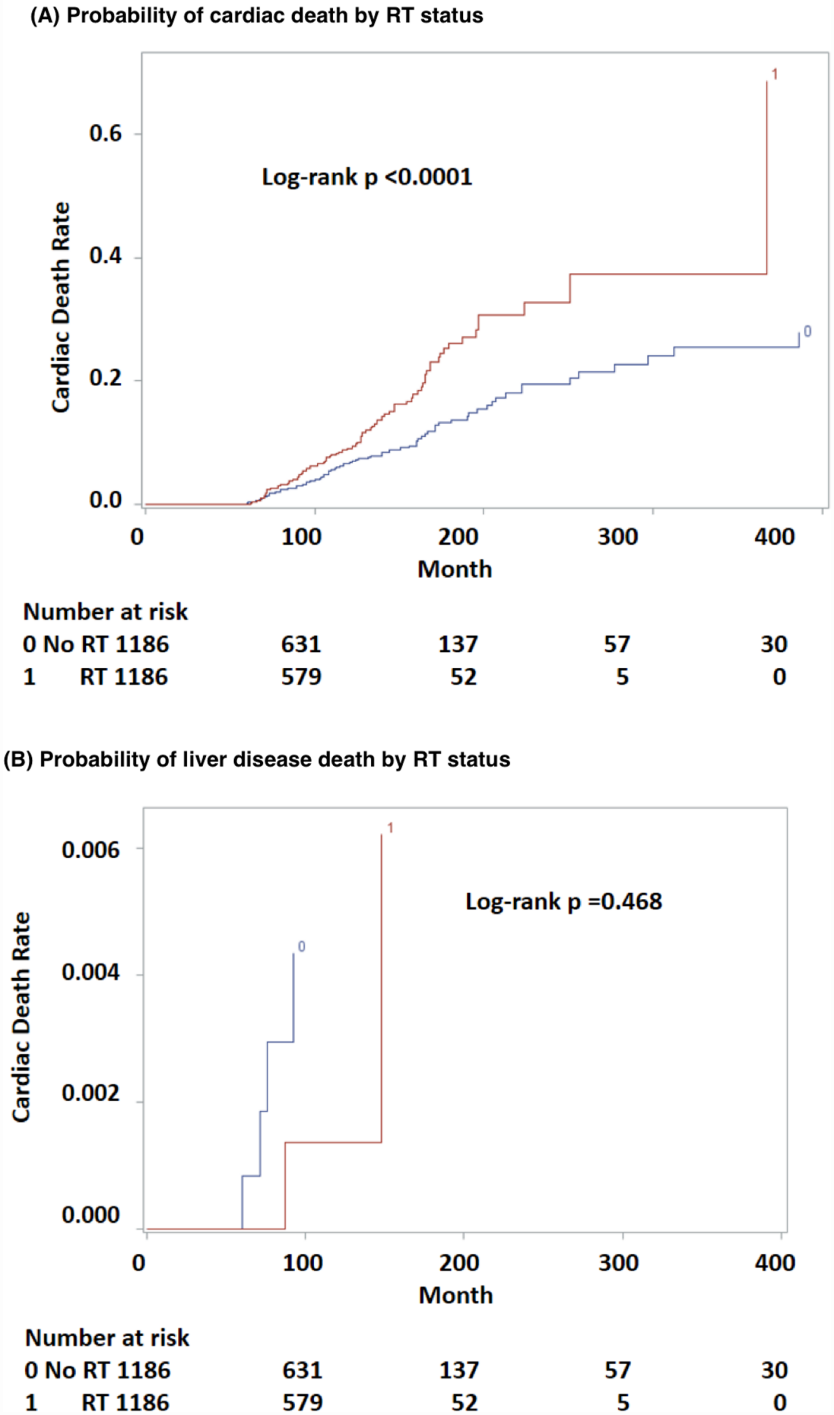
Probability of cardiac death (A) and liver disease death (B) by RT status for long-term survivors of esophageal cancer in propensity score matched data.

To control for as many variables as possible while examining differences between RT and no-RT, propensity score matching was performed. Patient populations were satisfactorily matched by propensity score with no statistically significant differences in various categories between groups (Table 2). In this propensity score matched data, patients who received RT continued to have a higher risk of cardiac death than those who did not (log-rank p < 0.0001, Fig 4A). As a control, the effect of RT on liver disease death was also evaluated. The results displayed no association of RT to esophagus and liver disease death (log-rank p = 0.468, Fig 4B). Cox proportional hazards regression models showed that receiving RT was significantly associated with increased risk of cardiac death as compared to not receiving RT in both univariate (HR = 1.751; 95% CI, 1.317–2.329) and multivariate (HR = 1.960; 95% CI, 1.469–2.615) models (Table 3). As compared to tumors located at 33–40 cm from the incisors, patient with tumors located at 15–24 cm or 25–32 cm from the incisors had a lower risk of having cardiac death (HR = 0.538; 95% CI, 0.333–0.870 and HR 0.684; 95% CI, 0.473–0.990 respectively). Additionally, younger patients and more recently diagnosed disease were associated with less risk of cardiac disease (HR = 1.069; 95% CI, 1.053–1.085 and HR 0.960; 95% CI, 0.942–0.977 respectively) as shown in Table 3.

**Table 2:** 1:1 matched patient population by propensity score.

	RT N (%)	No RT N (%)	P Value (Chi-square)
	N = 1186	N = 1186	
Mean propensity score ± SD	0.496 ± 0.219	0.496 ± 0.219	
Age (year)			
Median	63	63	
≤ 55	287	296	0.804
56–65	404	389	0.375
66–75	365	359	0.635
> 75	130	142	
Race			
White	1008	1024	0.322
Non-White	178	162	
Gender			
Male	899 (76)	904 (76)	0.779
Female	287 (24)	282 (24)	
Histology			
SCC	479 (40)	483 (41)	0.901
AC	707 (60)	703 (59)	
Site			
15–18	28 (2)	22 (2)	0.385
19–24	98 (8)	85 (7)	0.251
25–32	198 (17)	211 (18)	0.674
33–40	752 (63)	759 (64)	
Unknown	110 (9)	109 (9)	0.876
Stage			
Local	762 (40)	755 (71)	0.627
Regional	424 (40)	431 (17)	
Year of diagnosis			
1973–1992	232 (20)	271 (23)	0.106
1993–2002	495 (42)	462 (39)	0.556
2003–2007	459 (39)	453 (38)	
Surgery			
Yes	901 (76)	902 (76)	0.925
No	285 (24)	284 (24)	
Cardiac death			0.110
Yes	115 (10)	93 (8)	
No	1074 (90)	1093 (92)	
Pulmonary death			0.611
Yes	53 (4)	48 (4)	
No	1133 (96)	1138 (96)	
Liver death			0.157
Yes	2 (0.2)	6 (1)	
No	1184 (99.8)	1180 (99)	
Total death			0.234
Yes	589 (50)	560 (47)	
No	597 (50)	626 (53)	

RT, radiotherapy; SCC, squamous cell carcinoma; AC, adenocarcinoma.

**Table 3:** Cox proportional hazards model in the propensity score matched data.

	Model 1: Univariate		Model 2: Multivariate Including all factors		Model 3: Multivariate (Selection = Stepwise)	
	HR (95% CI)	P*	HR (95% CI)	P*	HR (95% CI)	P*
RT: Yes/No	1.751 (1.317–2.329)	**<0.0001**	1.961 (1.466–2.624)	**<0.0001**	1.960 (1.469–2.615)	**<0.0001**
Age: every year increase	1.060 (1.046–1.075)	**<0.0001**	1.068 (1.052–1.085)	**<0.0001**	1.069 (1.053–1.085)	**<0.0001**
Race: Non-White /White	0.801 (0.545–1.177)	0.259	0.890 (0.590–1.344)	0.5796		
Gender: Male/Female	0.976 (0.718–1.327)	0.877	1.278 (0.915–1.786)	0.1506		
Histology: AC/SCC	0.994 (0.751–1.315)	0.964	1.119 (0.781–1.604)	0.5387		
Site: Unk/33-40 cm	1.006 (0.627–1.615)	0.981	0.706 (0.426–1.171)	0.1780	0.753 (0.465–1.219)	0.2482
Site: 25-32/33-40 cm	0.879 (0.610–1.268)	0.492	0.743 (0.495–1.117)	0.1532	0.684 (0.473–0.990)	**0.0443**
Site 15-24/33-40 cm	0.760 (0.473–1.221)	0.257	0.549 (0.331–0.910)	0.0202	0.538 (0.333–0.870)	**0.0115**
Stage: Regional/Local	0.840 (0.626–1.126)	0.244	0.874 (0.643–1.186)	0.3869		
Year: every year increase	0.970 (0.953–0.988)	**0.0008**	0.955 (0.936–0.974)	**<0.0001**	0.960 (0.942–0.977)	**<0.0001**
Surgery: Yes/No	0.651 (0.481–0.879)	**0.005**	0.799 (0.565–1.130)	0.2048		

Bolded values are statistically significant. HR, hazard ratio; RT, radiotherapy; AC, adenocarcinoma; SCC, squamous cell carcinoma; Unk, unknown.

## Discussion

Prospective randomized controlled trials have demonstrated disease-free and overall survival benefits with the addition of RT to multimodality therapy for esophageal cancer [14,15,16]. However, long-term benefits may be diminished by cardiac toxicity. Using a large cohort of esophageal cancer survivors as well as propensity-matched analysis, we posit that RT may be an associative—not necessarily causal—factor with cardiac mortality in esophageal cancer survivors, a finding that has been consistent with multiple other inferences [17,18,19,20,21,22] Although these data are not applicable to non-survivors, long-term survivors (as defined, surviving at least five years after treatment) are more common than in the past due to advances in cancer treatment, thus these findings are applicable to a broader patient population in today’s day and age.

Though the use of population-based analyses provides high numbers of esophageal cancer survivors with which to examine associations, there are several caveats that cannot be ignored. First, this study strictly analyzed the outcome of interest in long-term esophageal cancer survivors, defined as those patients that have survived for five years and thus have reached a minimum risk of further cancer-related mortality. Next, there was no way to quantify the actual cardiac RT doses/techniques in each patient, and such analysis would require careful review of individual patient RT plans and treatment records. This imprecision does not invalidate the overall findings, but rather necessitates further study to better qualify and quantify this risk. Additionally, the SEER database does not provide information on comorbidities, which could substantially influence the conclusions of this and similar analyses [22]. It is certainly possible that the younger patients in the RT group could have lived longer to manifest more cardiac deaths, although the higher incidence of regional/distant disease in this group could counteract this impression. With these interesting findings, the natural next phase of this study will be to move to the SEER-Medicare or National Cancer Data Base to incorporate these comorbidities. Finally, the RT group was less likely to undergo surgery; whether this related to greater preexisting cardiopulmonary comorbidities precluding surgery cannot be proven, although receipt/non-receipt of surgery was not associated with cardiac death in secondary statistical analysis. To this extent, a notable strength of this work is the use of propensity-matched data; though comorbidities could not be “matched”, many other potentially confounding variables (including age and receipt of surgery) were matched as much as possible, with a substantial association between RT and late cardiac death still present.

Regarding our findings, the RT group had greater proportions of SCC and hence proximal esophageal disease, which is anatomically farther from the heart (or ventricles). This suggests (albeit in a speculative fashion) that radiation of ACs (most commonly in the lower esophagus) could provide even higher cardiac doses than the RT group likely experienced in this study, and hence potentially increase cardiac morbidity/mortality if a causal relationship is indeed proven. It is known that RT doses are predictive of symptomatic pericardial effusions in esophageal cancer patients [23,24] but proving causation of a late toxicity in esophageal cancer needs further high-quality evidence.

Despite this dearth of data, there have been many studies examining the detrimental effects of RT on the heart, and hence our study’s associative findings are not without several precedents. Radiation’s effects on the heart were first postulated in the early 1960s [25], Decreases in cardiac ejection fraction during RT for esophageal cancer have been well-documented [26,27] and cardiac damage can readily be assessed through the use of myocardial perfusion imaging [28,29], positron emission tomography [30], single photon emission computed tomography [31], and magnetic resonance imaging [32,33]. Additionally, functional changes can be appreciated clinically, such as decreased blood pressure, increased heart rate, and decrease in heart volume [34].

Techniques to reduce cardiac dose in hopes of reducing these detrimental effects on the heart have been attempted. Breast cancer data has shown that patient positioning could be effective, as well as judicious treatment of elective nodes and RT technique [35]. It follows, then, that there can be other techniques by which to reduce cardiac doses in esophageal cancer RT. Whereas esophageal cancer has been traditionally treated with three-dimensional conformal technique, the use of intensity-modulated RT (IMRT) has been shown to decrease cardiac doses and potentially prevent associated long-term complications, albeit in retrospective data [36,37]. It is possible that in these data, the probability of cardiac death decreasing with diagnosis year could be associated with increased use of new technologies such as IMRT. The use of even more advanced techniques such as arc therapy have further illustrated cardiac RT dose reductions from IMRT [38]. Lastly, the nascent frontier of proton RT for various cancers is growing rapidly, and could provide clinically meaningful improvements in acute and long-term toxicities in esophageal cancer [39]. An analysis of dose-volume histograms comparing proton versus traditional x-ray therapy has shown that protons can reduce the irradiation dose and volume of heart irradiation in esophageal cancer, potentially decreasing complications. [40] Furthermore, the worldwide expansion of carbon ion RT has also generated a phase I/II trial demonstrating its safety and efficacy in esophageal cancer [41]. Although this trial did not examine cardiac toxicities, a recent dosimetric study of cardiac doses in patients with mediastinal Hodgkin lymphoma treated with protons versus carbon ions demonstrated decreased cardiac doses with the latter, potentially owing to a smaller lateral penumbra [42]. A major question for clinicians to now address is which patients benefit most from these new (and expensive) technologies to spare cardiac dose—in other words, the patients most likely to be long-term survivors may be the same patients for which these new technologies may be most cost-effective [43].

## Conclusions

In this population-based analysis of survivors of esophageal cancer, RT may be associated with increased risk of cardiac death, which is in concordance with long-term outcomes in irradiated left-sided breast cancer and Hodgkin’s lymphoma patients. Though not without limitations, the use of propensity matching in this study provides added evidence to the burgeoning notion that cardiac doses in esophageal cancer radiotherapy indeed have important clinical consequences in survivors. High-quality, long-term studies are undoubtedly needed in order to validate these notions.
